# When Rare Is Not Small: Amyotrophic Lateral Sclerosis Initiatives and Therapy

**DOI:** 10.1002/exp2.70114

**Published:** 2026-02-13

**Authors:** Yang Liu, Xue Xia, Yingfang Chen, Haoying Yang, Xiaochen Chen, Lei Cai, Bingyang Shi

**Affiliations:** ^1^ The Zhongzhou Laboratory for Integrative Biology, Henan International Joint Laboratory of Nanobiomedicine, School of Life Sciences Henan University Kaifeng Henan China; ^2^ The Second Clinical Medical School Henan University Kaifeng China; ^3^ School of Biomedical Engineering, Faculty of Engineering and Information Technology University of Technology Sydney Sydney Australia; ^4^ Beijing AskHelpU Medical Tech Co. Ltd. Beijing China

## Abstract

In the precision‐medicine era, rare diseases must not be sidelined in translational infrastructure. The Mr. Cai Lei—led “Ice‐Breaking Team” turns an amyotrophic lateral sclerosis patient community into a sustainable ecosystem, realigning philanthropy, data, and research and development to reshape rare‐disease pipelines and guide precision therapies, offering a replicable blueprint for rare‐disease strategies.

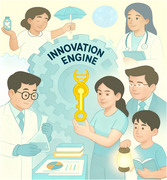

## 1 Charting a Path Forward for Rare Disease Treatment Through Amyotrophic Lateral Sclerosis “Ice‐Breaking” Models

Rare diseases, though individually uncommon, collectively affect millions of people worldwide, constituting a substantial yet often underserved patient population. Owing to their low prevalence per condition, rare diseases frequently fall outside the strategic priorities of major pharmaceutical companies, limiting the pace and scope of therapeutic development. At the same time, personalized medicine is becoming the norm, enabled by genomics, biomarkers, and targeted delivery. If we can tailor therapies to subgroups of common diseases, the same logic has no reason not to apply to rare diseases. The challenge extends beyond scientific innovation, but structural barriers: fragmented patients, scarce funding, and weak incentives.

Our discussion draws on the model pioneered by Mr. Cai Lei and the Ice‐Breaking Team in China, who combined philanthropy, public engagement, and an open laboratory network to accelerate translational research for amyotrophic lateral sclerosis (ALS). Their approach highlights how new funding mechanisms, public awareness and cross‐sector collaboration could reshape the rare disease research and development (R&D) landscape. Ultimately, solving rare diseases requires building a sustainable ecosystem, one that balances ethics with access, protects innovation while ensuring affordability, and fosters a scientific common that turns limited resources into an “infinite arsenal” against disease.

## 2 Rarity Is Not the Barrier: Reframing Rare Diseases in Precision Medicine

**FIGURE 1:**
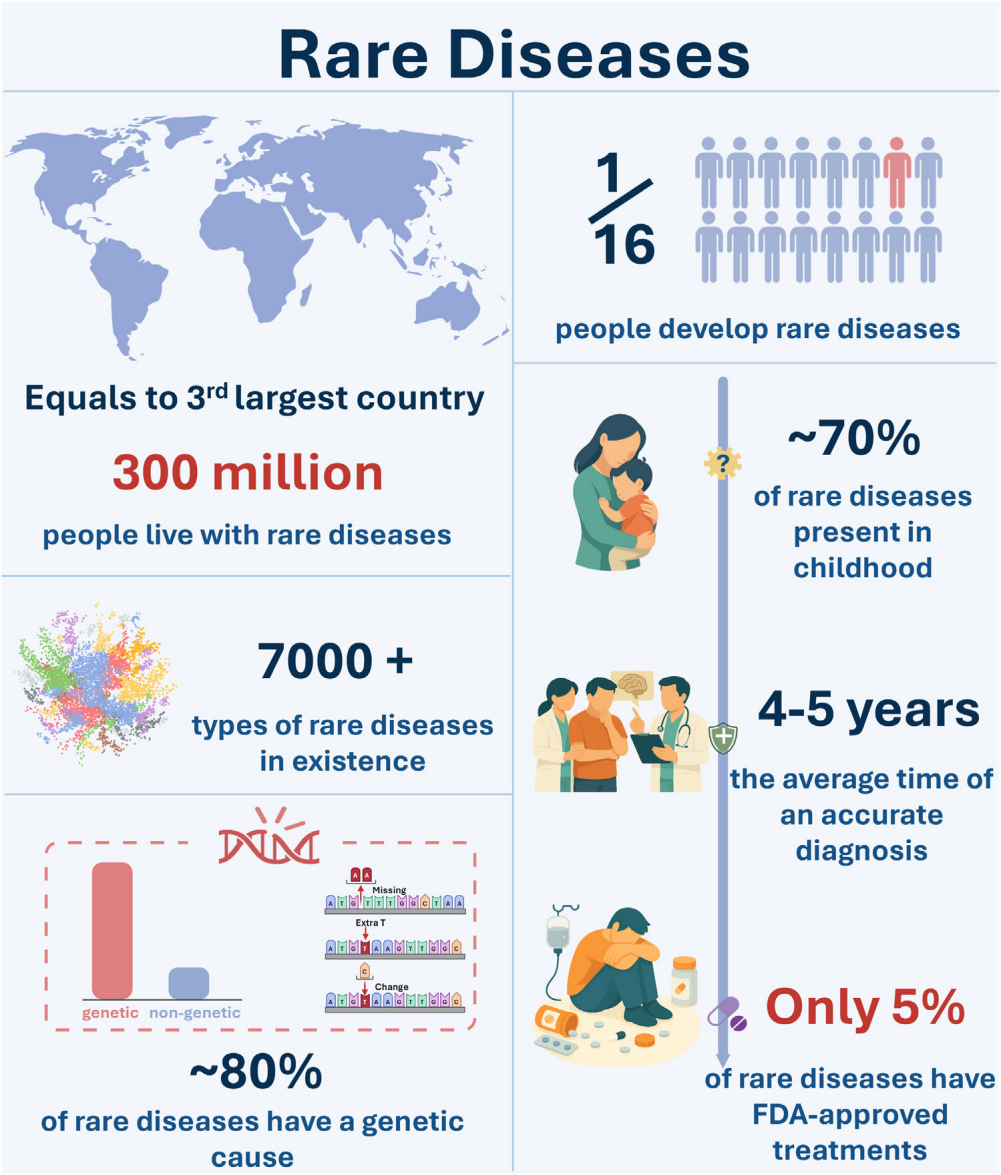
Rare disease landscape. The collective burden of rare diseases affects a considerable population worldwide, and their rarity should not impede technology platform innovations in the era of precision medicine.

Rare diseases, each affecting a small number of individuals, collectively impact more than 300 million people worldwide [1, 2]. This burden, comparable in magnitude to many common diseases, demonstrates that the global burden of rare conditions is far from marginal (Figure 1). Despite this, therapeutic development for rare diseases remains disproportionately underfunded and underprioritized. The core challenge lies in their fragmented prevalence: most rare diseases affect fewer than 1 in 2000 people, and many impact just a handful of individuals worldwide [3, 4]. This epidemiological reality leads to limited commercial incentives, especially under traditional drug development models where return on investment is tightly coupled to patient volume.

Consequently, rare disease therapies have historically relied on targeted public funding, orphan drug legislation, or philanthropic efforts to progress [5]. Large pharmaceutical companies may engage in rare disease programs where platform technologies (e.g., antisense oligonucleotides, AAV vectors) can be reused, but they tend to concentrate their resources on diseases with broader market potential. This strategic calculus has reinforced a structural inequity: patients with rare diseases often face not only a lack of effective treatments, but also delayed diagnoses, clinical trial inaccessibility, geographic variations, and limited post‐market care options. Reflecting this urgency, the World Health Organization (WHO) has recently elevated rare diseases to a global health priority, urging member states to embed them within their national health plans, expand equitable access to diagnosis and treatment, including universal newborn screening, and commit to a 10‐year global action plan that coordinates research, policy, and care worldwide [6]. In line with this global agenda, multiple policy frameworks now support rare disease research and therapeutic translation. Many countries have established orphan‐drug incentives and flexible approval pathways, and comprehensive overviews of national landscapes can be accessed through platforms such as Rare Diseases International (RDI) Resource Maps and the International Rare Diseases Research Consortium (IRDiRC) Policies and Guidelines, while specific regulatory details can be found on each country's official agency websites.

In parallel, the rise of personalized medicine has transformed our understanding of disease heterogeneity and therapeutic response [7]. Driven by advances in genomics, transcriptomics, and biomarker discovery, the concept of “one‐size‐fits‐all” medicine is giving way to tailored treatment strategies, even within diseases previously considered homogeneous. Oncology and immunology, for example, have embraced molecular subtyping to guide therapy selection, enabling more precise and effective interventions [8].

However, while personalized medicine shares philosophical alignment with rare disease treatment, both aim to address unique molecular profiles, the translational infrastructure supporting rare diseases has not advanced at the same pace. In practice, the individualized nature of rare diseases has not yet translated into equitable therapeutic development. Instead of enabling innovation, rarity often remains a barrier due to high per‐patient development costs, regulatory complexity, and insufficient longitudinal data to support traditional drug approval frameworks.

This disconnect highlights the urgent need for foundational technological and systemic innovations. Platform technologies, such as modular gene editing systems [9], programmable RNA drugs [10], synthetic biology circuits [11], or nanoparticle‐based delivery systems [12], offer the potential to generate reusable, scalable therapeutic solutions. At the same time, harmonizing regulatory pathways, enabling adaptive trial designs, and embedding patient registries into research pipelines can reduce both risk and cost. If implemented strategically, these advances can help shift the focus from rarity as a limitation to rarity as an opportunity for innovation, allowing the field to sustainably deliver therapies that are both clinically impactful and economically viable.

## 3 Gene Therapy Beyond the Viral Vector: A Shift Toward Scalable Innovation

Gene therapy has emerged as a key strategy for rare disease treatment, particularly for monogenic disorders [5]. While viral vectors, especially adeno‐associated viruses (AAVs), have driven early clinical successes (Zolgensma, Luxturna), the field is now facing a strategic turning point. Several leading pharmaceutical companies have recently scaled back or terminated AAV‐based programs, citing challenges including immune toxicity, limited tissue penetration, high manufacturing costs, and uncertain long‐term outcomes.

This withdrawal reflects deeper structural issues. AAV delivery to the central nervous system (CNS) often requires high systemic doses, increasing the risk of hepatotoxicity and immune responses. Re‐dosing is complicated by the development of neutralizing antibodies, and achieving consistent biodistribution, especially in heterogeneous tissues like the brain, remains technically difficult. For rare diseases, the economic return on investment is often insufficient to justify these risks, even with orphan drug incentives. Furthermore, some high‐profile failures in late‐stage trials have shaken investor and regulatory confidence, leading companies to reprioritize toward broader or lower‐risk pipelines.

**FIGURE 2:**
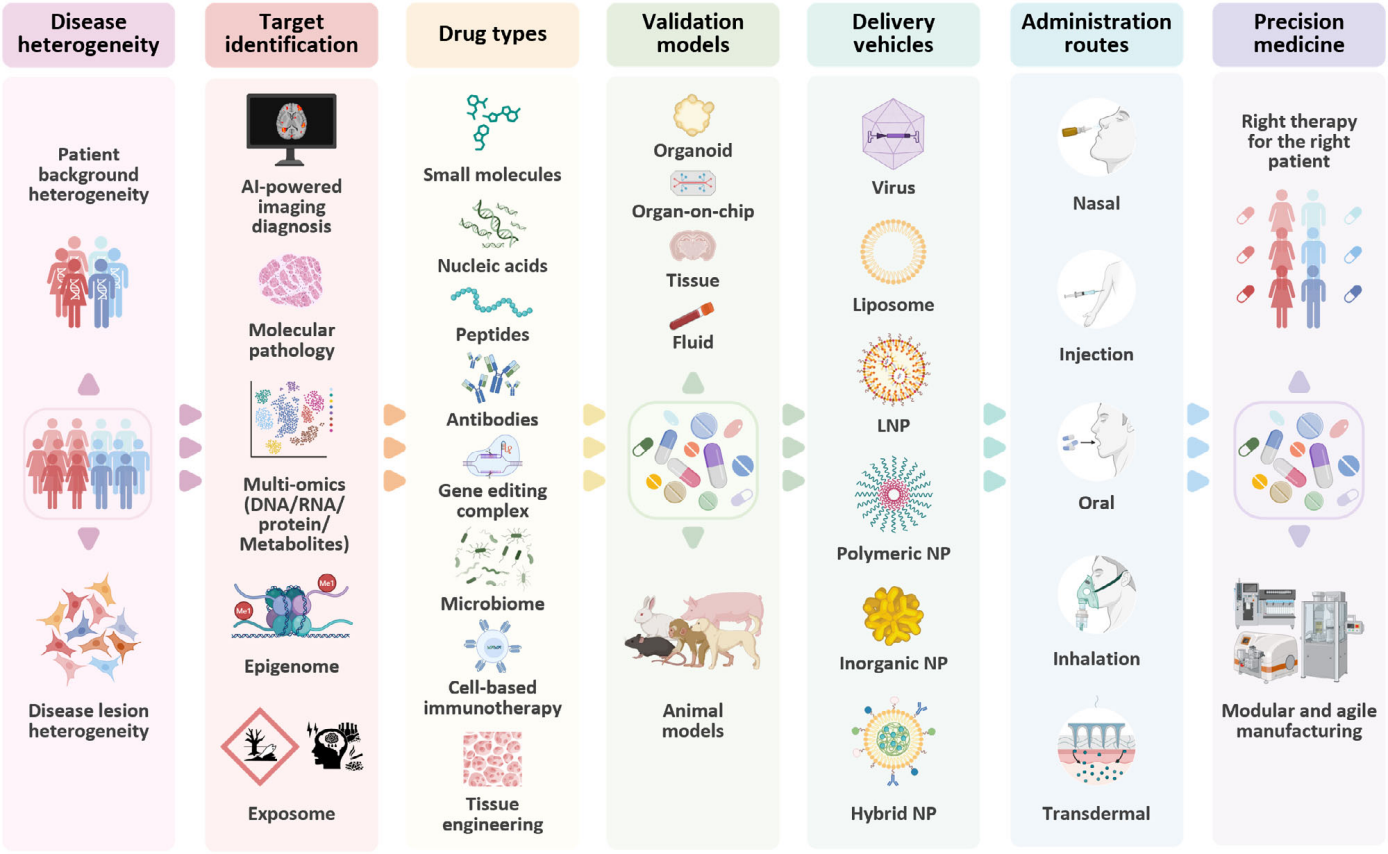
Precision therapy rationale, approaches, and tools. The technological innovations driving this shift are shared with more prevalent diseases that also require precision medicine, reinforcing the idea that disease rarity should never dilute innovation. Abbreviation: Artificial Intelligence (AI); Deoxyribonucleic Acid (DNA); Ribonucleic Acid (RNA); Lipid nanoparticle (LNP); Nanoparticle (NP).

Genetic background heterogeneity and cellular‐level variability in disease lesions demand a truly integrative approach to target identification, one that harnesses artificial intelligence (AI)‐powered imaging, pathological biomarkers, and multi‐omics spanning the transcriptome, proteome, metabolome, epigenome, and even environmental “exposome” data. This convergence with AI opens the door to building virtual cells, computationally modeled simulation systems that could revolutionize hypothesis testing and drug discovery. These AI‐driven frameworks can also condense heterogeneous patient‐level information into evolving, clinically meaningful digital phenotypes, providing a generative synopsis of multi‐source data for target prioritization, risk stratification, and trial design. We are witnessing a rapid expansion of innovative therapeutic modalities, ranging from single‐molecule constructs to nanoscale engineering and subcellular‐ to micrometer‐scale designs, including stem‐cell‐based tissue engineering solutions. However, validating these approaches remains a thorny challenge. Traditional animal models often fall short of faithfully projecting human biology, while organoids, organ‑on‑chips, and tissue‑fluid systems still lack crucial in vivo parameters, such as circulatory dynamics, hormonal interplay, and inter‐organ exchanges that define real‐life disease contexts. On the delivery front, both viral and non‐viral vectors continue to improve, especially following recent U.S. Food and Drug Administration (FDA) approvals of lipid‐based nanoparticle therapies. Lipid nanoparticles (LNPs), for instance, are undergoing a transformation through “programming” strategies that enhance targeting, stability, and payload delivery; think of them as “smart missiles” in gene therapy. Complementing vector development, breakthroughs in formulation, administration routes, and manufacturing processes are beginning to make truly personalized treatments for rare diseases a feasible reality (Figure 2).

These developments raise several open questions for the field:

- Can we overcome the immunogenicity and dosing barriers of viral vectors (repeat dosing despite pre‐existing neutralizing antibodies with low hepatic toxicity), especially for CNS indications?
- What regulatory and trial design reforms are needed to support small‐population, high‐uncertainty interventions?
- How can manufacturing be made more flexible and cost‐efficient across multiple gene therapy products?
- Will non‐viral platforms offer sufficient delivery & transfection efficiency and durability to replace viral vectors in high‐barrier tissues?

Addressing these questions will require both scientific innovation and structural reform. It also underscores the need to explore alternative development models that can de‐risk translation while maintaining a focus on patient impact, an area where community‐driven approaches, such as those led by an ALS patient and advocate, Mr. Cai Lei, offer valuable insight.

## 4 The “Ice‐Breaking” Model: A Patient‐Centered Framework for Rare Disease Innovation

**FIGURE 3:**
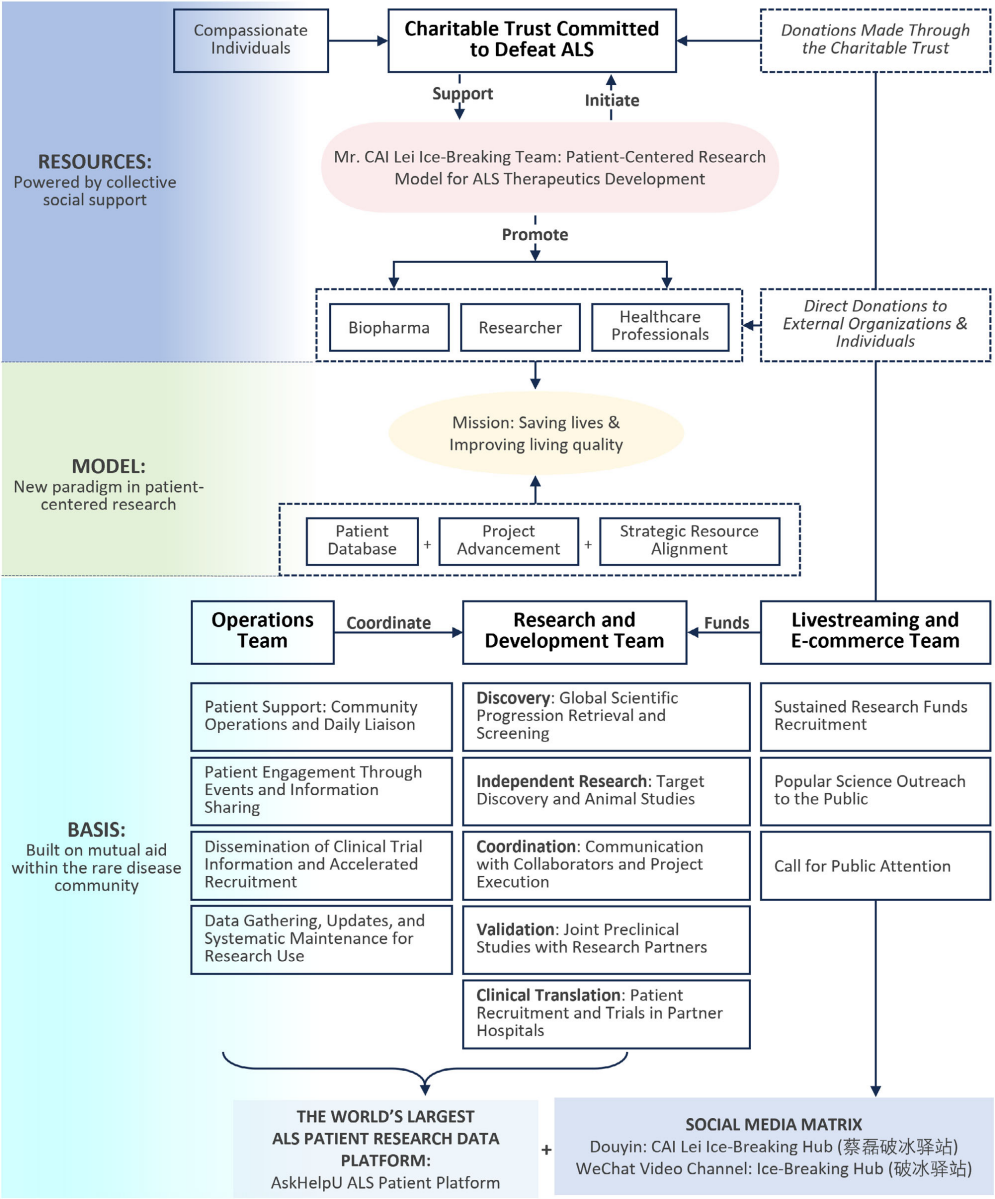
Collaborative structure of the “Ice‐Breaking Team” initiated by Mr. Cai Lei. An innovative philanthropic model that integrates social support, patient‐centered research, and cross‐platform R&D collaborations with shared funding resources. The Ice‐Breaking Team is a non‐profit integrated platform initiated by a group of ALS patients, encompassing patient communities, basic research, drug development, and philanthropy. It provides a valuable reference model for rare disease research and charitable initiatives. Abbreviation: Amyotrophic Lateral Sclerosis (ALS).

In the face of mounting scientific, economic, and translational challenges in rare disease therapy, patient‐led initiatives have begun to reshape the research and development landscape. Among the most prominent is the work of Mr. Cai Lei, an ALS patient, entrepreneur, and founder of the “Ice‐Breaking Team”, a collaborative, multi‐stakeholder initiative (Figure 3) that aims to accelerate therapeutic discovery and translation for ALS and other neurodegenerative diseases and rare diseases.

The Ice‐Breaking Team's approach is distinguished by its integration of patient needs, scientific collaboration, and translational infrastructure. Central to this effort is the “AskHelpU” platform, a Chinda‐based initiative that has grown into the world's largest ALS patient engagement network. This platform functions not only as a data repository but also as a clinical resource, enabling patient stratification, rapid trial recruitment, and real‐world needs assessment. It exemplifies how empowered patient communities can contribute directly to the translational ecosystem.

What sets the Ice‐Breaking model apart is its adaptive, project‐based collaboration strategy. The team actively scouts promising research directions through global literature tracking, academic dialogue, and early‐stage biotechnology outreach, and then initiates targeted collaborations with universities, clinical centers, and companies. Rather than passively funding academic research, the team co‐designs projects, participates in protocol development, and embeds team members within preclinical and clinical workflows. This hands‐on involvement helps bridge the often‐fragmented divide between discovery and application.

Each therapeutic development effort under the Ice‐Breaking framework follows a four‐stage process:

**Table:** 

Target identification: Mining scientific literature and international pipelines for promising biological targets or therapeutic modalities.
Collaborative matchmaking: Establishing joint research agreements with laboratories, biotech firms, or contract research organizations (CROs) to initiate preclinical work.
Proof‐of‐concept execution: Supporting in vitro and in vivo validation, with team members overseeing progress on‐site or remotely.
Translational acceleration: Utilizing their rapid‐recruitment patient platform, regulatory guidance, and media engagement to initiate investigator‐led clinical studies.

This model has already supported the development of early‐phase therapies, including RAG‐17, a gene‐silencing candidate targeting *SOD1*‐linked ALS, now in clinical evaluation [13]. It has also demonstrated how alternative funding sources, from philanthropic livestreams to patient‐family investment syndicates, can substitute for traditional venture capital or pharmaceutical partnerships in the earliest stages of development.

By coupling scientific rigor with mission‐driven urgency, the Ice‐Breaking Team represents a novel blueprint for rare disease innovation. Rather than waiting for large‐scale industry engagement, this model builds translational capacity around the patient, drawing together academic, clinical, and commercial actors in a task‐oriented and time‐sensitive manner.

## Interview Section

### Interview With Mr. Cai Lei: Perspectives From the Frontline of ALS and Rare Disease Research

To address the pressing questions that patients, families, researchers, and policymakers care about, the *Exploration* Editorial Team held an in‐depth conversation with Cai Lei's team. Mr. Cai is a well‐known entrepreneur living with ALS and the founder of the Ice‐Breaking initiative and the AskHelpU ALS Patient Platform in China. Mrs. Rui Duan, co‐founder of the Ice‐Breaking Hub with her husband, Mr. Cai, and director of its livestream channel, transitioned from her previous professional career to devote herself fully to fundraising coordination, public engagement, and patient advocacy. With the support of Ms. Yingfang Chen from the highly engaged and mission‐driven Ice‐Breaking Team, they offered thoughtful and grounded reflections drawn from Mr. Cai's experience leading this collective effort.

## Section 1: The Clinical Reality and Real‐World Patient Needs

***Exploration**:* What do ALS patients most urgently need? Not limited to research—do they, for example, need standardized psychological care? And compared with more common diseases, what are the biggest gaps in the ALS R&D ecosystem?

**Mr. Cai Lei and Ice‐Breaking Team**:


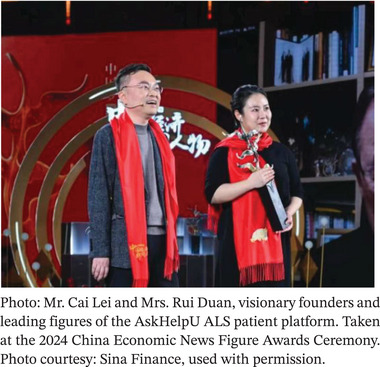


ALS is a burdensome neurodegenerative rare disease. In China, the incidence is about 1.6 per 100,000, with around 100,000 patients nationwide. Once diagnosed, patients face progressive muscle wasting and paralysis, eventually losing the ability to speak, swallow, and even breathe. Average survival is just 2 to 5 years. It is often described as “the deadliest of the world's five terminal diseases.” Despite 2 centuries of effort, the cause remains unclear, therapeutic targets are undefined, and no treatment can halt the disease. The cure rate is still zero.

For ALS patients, the most urgent need is a breakthrough in drug development—effective therapies that can significantly slow or halt disease progression, or even achieve a cure. This is also the goal our team continues to pursue. However, while waiting for such breakthroughs, patients endure not only physical suffering but also tremendous psychological pressure. Effective caregiving can, to some extent, prolong survival. Our “AskHelpU (渐愈互助之家),” meaning Home of the Gradual Recovery Mutual Aid, is a community that offers patients a platform to communicate, share caregiving knowledge, and exchange practical experiences. At the same time, we conduct surveys within the patient community to summarize and disseminate effective practices. Beyond physical challenges, patients often fall into anxiety, depression, and other negative emotions, which makes it essential to establish standardized psychological support programs. We are currently working with the Chinese Mental Health Association to explore this field, for example, by arranging regular sessions with professional counselors for patients and their families, and by organizing peer exchange activities. These efforts help patients encourage one another, share experiences, and strengthen their confidence and courage in facing the disease.

There are more than 7000 rare diseases worldwide, and currently, about 95% of them still lack effective treatments. In the field of rare diseases represented by ALS, the biggest gaps in the research and healthcare ecosystem include:

1. Weakness in basic research
  - Unclear pathogenesis: For rare diseases such as ALS, the exact pathological mechanisms remain poorly understood. Motor neuron degeneration may involve multiple complex factors, including oxidative stress, protein misfolding, mitochondrial dysfunction, and immune system abnormalities, but how these factors interact and trigger disease onset and progression is still unknown. This makes it extremely difficult to identify effective therapeutic targets and hinders subsequent drug development efforts.
  - Limited research samples: Because patient numbers are relatively small, available clinical samples (such as patient tissue samples, especially brain and spinal cord tissue, and biomarker data) are scarce. Moreover, these samples are scattered across different regions and medical institutions, making it difficult to consolidate them for large‐scale, high‐quality studies. This greatly limits deeper and more comprehensive exploration of the disease.
2. Insufficient funding
  - Low commercial attractiveness: The market for rare disease drugs is relatively small. From a commercial standpoint, pharmaceutical companies often prefer to invest in diseases with broader patient populations and higher returns. For rare diseases such as ALS, developing a new drug faces high costs and high risks but limited expected returns, which reduces external investment willingness and creates a significant funding gap for drug R&D.
  - Limited research grants: Although governments and philanthropic organizations provide some funding, the amount allocated to rare disease research remains much lower than that for common diseases. This is far from sufficient to support comprehensive research activities across all stages, from basic science to clinical trials.
3. Major challenges in clinical trials
  - Patient recruitment difficulties: Clinical trials require large numbers of eligible patients to validate the safety and efficacy of drugs. However, the small patient population makes it extremely difficult to recruit enough participants at the appropriate stage of disease, leading to slow trial progress or even trial failure, thereby prolonging drug development timelines.
  - Complex trial design: Rare disease patients often show significant heterogeneity—the severity and progression of illness vary greatly across individuals. This makes it difficult to standardize criteria and accurately assess drug efficacy in trials. In addition, the short survival time of many patients raises ethical concerns and further complicates study design and interpretation, placing higher demands on the scientific rigor and feasibility of trials.
4. Unequal distribution of medical resources
  - Shortage of specialized teams: The number of physicians who are well‐versed in rare disease diagnosis, treatment, and long‐term management is limited. Many primary‐level medical institutions lack relevant expertise, which may lead to misdiagnosis, missed diagnosis, or difficulties in providing comprehensive and evidence‐based treatment recommendations.
  - Insufficient multidisciplinary collaboration: Diagnosis and treatment of rare diseases typically require multidisciplinary cooperation, involving neurology, rehabilitation, pulmonology, nutrition, and other specialties. At present, however, collaboration mechanisms across disciplines remain underdeveloped, with poor information flow and limited integration, making it difficult to deliver comprehensive and efficient care.

***Exploration**:* Do you believe international cooperation can promote progress in ALS treatment? In terms of patient care, scientific research, and medical guidelines, has the current level of international cooperation reached an acceptable or satisfactory state?

**Mr. Cai Lei and Ice‐Breaking Team**:

Treating ALS and other rare diseases requires broad international collaboration, bringing together researchers, medical institutions, and pharmaceutical companies worldwide. Compared with the past, the level of international cooperation has improved significantly. In 2024, with the support and advocacy of former United Nations (UN) Secretary‐General Ban Ki‐moon, we deepened global scientific cooperation, establishing close ties with more than 70 distinguished professors and their elite teams, as well as over 60 specialized research institutions and biopharmaceutical companies. Some of these collaborations have already advanced to a deeper stage.

On the research front, we have connected with internationally renowned experts such as Prof. Don W. Cleveland from the University of California, San Diego, an ALS research expert, and Prof. Merit Cudkowicz, Chair of Neurology at Massachusetts General Hospital, to continuously explore new directions in drug development and therapeutic strategies. In terms of medical guidelines, we have engaged with patient organizations such as AnswerALS, one of the most comprehensive ALS research projects ever assembled, to learn from their experiences in healthcare and patient support. We have also partnered with international ALS organizations and patients to host events that raise global awareness. For example, in April 2024, Mr. Cai Lei, together with Mr. Jaime Lafita, founder of the Spanish nonprofit DalecandELA, and the US‐based ALS Network, launched the “Crossing Death Valley” challenge, a transcontinental cycling initiative spanning three continents. The aim was to unite more scientists, clinicians, and people from all walks of life to bring greater attention to ALS patients and the urgency of life‐saving treatments, thereby increasing the realistic possibility of overcoming ALS through global collaboration.

However, international cooperation has not yet reached a satisfactory level. Key obstacles remain:

1. Differences in policies and regulations: Each country has its own regulatory framework, and requirements for clinical trial approval and drug supervision vary greatly. This raises the difficulty and cost of cross‐border cooperation and slows drug development.
2. Barriers to data sharing: Strict confidentiality regulations create hesitation in exchanging research data across borders. ALS research requires extensive clinical and genetic data, yet many countries impose stringent rules on data to protect privacy.
3. Complex financial oversight: Funding sources and expenditures in international collaborations are constrained by different national financial regulations. On the one hand, fundraising efforts may face hurdles due to varying tax policies and charitable laws, affecting efficiency and scale. On the other hand, once raised, funds are subject to different regulatory standards, creating obstacles for cross‐border flow and utilization.

Effective collaboration will benefit from clearer guidelines on the implementation of these necessary regulations.

## Section 2: Translational Medicine Route and Strategy

***Exploration**:* In your opinion, what are the core bottlenecks in translating ALS research from the “laboratory” to “clinical application”? Is it the unclear pathogenesis, patient heterogeneity, drug delivery, or clinical trial design? In terms of short‐, mid‐, and long‐term challenges, which represent the most critical rate‐limiting steps?

**Mr. Cai Lei and Ice‐Breaking Team**:

In the short term, drug delivery and clinical trial design represent the most critical rate‐limiting factors. Even with some preliminary insights into disease mechanisms, achieving effective drug delivery to damaged motor neurons remains a major technical challenge, particularly given the need to cross the blood–brain barrier, which is essential for treating CNS disorders. At the same time, current clinical trial designs struggle to accommodate patient heterogeneity. For example, patient stratification is often imprecise, leading to therapeutic effects being masked by noise. Riluzole, the only widely recognized ALS drug, illustrates this issue: its benefits are modest and may only apply to certain patient subgroups, but existing trial designs cannot reliably identify such subpopulations. The lack of robust biomarkers further exacerbates this problem. Short‐term solutions include developing advanced delivery technologies (e.g., nanoparticle‐based carriers) and implementing biomarker‐driven stratification strategies (such as using neurofilament light chain, NfL) to improve trial efficiency and increase the probability of therapeutic success.

In the medium term, the central bottleneck lies in the incomplete understanding of pathogenesis, which is closely linked to patient heterogeneity. ALS arises from complex causes, including genetic mutations (e.g., *SOD1, C9orf72*), protein misfolding (e.g., TDP‐43 aggregates), neuroinflammation, and oxidative stress. However, a unified pathological model remains absent. Due to wide variation in age of onset, disease progression, and symptom presentation, existing research samples lack representativeness, resulting in a fragmented understanding of core mechanisms. As a rare disease, ALS faces particular challenges in assembling large, multi‐dimensional datasets. Many drug candidates that show efficacy in animal models have failed in clinical trials due to mechanistic uncertainty and interference from heterogeneity. Similar issues exist in Alzheimer's disease, but the rarity and variability of ALS make the problem even more acute. Medium‐term solutions will require substantial investment in basic research and the application of multi‐omics approaches (e.g., genomics, proteomics) to disentangle disease mechanisms.

In the long term, the greatest rate‐limiting factor lies in the economic and policy environment surrounding rare disease drug development. ALS therapeutics face the classic “orphan drug” dilemma: high cost and risk of development with limited market returns. This weakens pharmaceutical industry incentives and undermines the sustainability of academic research enthusiasm due to funding and translational barriers. Addressing this requires systemic policy measures, such as lowering thresholds for clinical research (e.g., streamlined approval processes), offering tax incentives, or providing research subsidies to encourage enterprise investment in rare disease therapeutics. The U.S. Orphan Drug Act provides a successful example, granting market exclusivity and financial support to stimulate rare disease drug development. Over the long run, progress will depend on establishing robust data‐sharing platforms (e.g., AnswerALS) and interdisciplinary ecosystems that combine AI‐driven analytics with long‐term patient cohorts. Such frameworks can gradually address the complexity and heterogeneity of ALS and foster a virtuous cycle between academia and industry.

***Exploration**:* Do you agree with the three‐stage strategy of “short‐term saving patients, medium‐term elucidating mechanisms, and long‐term pursuing a cure?” Should each stage be supported by different funding sources? What is your team's current strategy, and can government support be mobilized to strengthen rare disease research and treatment, for example, short‐term through medical insurance, and medium‐term through national research funding?

**Mr. Cai Lei and Ice‐Breaking Team**:

Our team endorses this three‐stage strategy. Short‐term— “Saving patients:” This stage does not rely on a single funding source. On one hand, for already‐approved drugs, expanding reimbursement coverage is key to improving patient access and affordability, leveraging public medical insurance funds and social healthcare systems. On the other hand, for drugs not yet formally approved but showing promising clinical benefit, patient‐driven models play an important role. Examples include the “family donation → researcher‐led trial → patient benefit” self‐rescue model, and “venture philanthropy,” in which patient groups and families invest directly in biotech companies, combining financial commitment and urgency for treatment with industry's technical expertise. Thus, short‐term patient care depends on multiple channels—public healthcare funds, patient/family resources, and innovative financial mechanisms.

Policy innovation is also crucial. Cell and gene therapies are among the most promising approaches for rare diseases. The Boao Lecheng International Medical Tourism Pilot Zone in Hainan has served as a national “test field” for health policy, leveraging a “first‐trial, first‐use” regulatory framework to accelerate the introduction of advanced international therapies. Under the Regulations on Promoting Biomedical New Technologies in the Pilot Zone of Hainan Free Trade Port, once a new biomedical technology is approved for clinical application in the zone, medical institutions must file prices with provincial health and insurance authorities for public disclosure. This ensures transparent, standardized pricing, protects patients' right to know, and enhances public trust. By aligning legal safeguards with innovation pathways, this framework has improved the efficiency of translational application and incentivized innovation. Given its positive impact, we hope such mechanisms can be extended nationwide, so that more patients can access cutting‐edge therapies like cell and gene therapy, while simultaneously promoting the development of China's healthcare industry.

Medium‐term—“Elucidating mechanisms:” The priority here is national research funding. Government‐supported programs can guide scientific communities to focus on rare disease mechanisms, such as the molecular pathways of disease onset and progression, and the identification of therapeutic targets. This is essential to breaking the bottlenecks that currently limit progress and will lay the groundwork for future cures.

Long‐term— “Pursuing a cure:” Achieving ultimate cures requires commercial capital. Industry has the ability to integrate resources, scale production, and bring therapies to market. Once the earlier stages of mechanism discovery and translational validation are in place, commercial investment can accelerate drug development, large‐scale manufacturing, and distribution, ensuring sustainable impact.

The role of government: Government can play a decisive enabling role across all three stages. Adjusting medical insurance policies, it can directly reduce financial burdens on families and improve access to therapies. By guiding research funding, it can fill gaps where market incentives are weak, ensuring resources are directed to key rare disease research. More broadly, governments can introduce tax incentives, R&D subsidies, and policy frameworks that encourage active participation by pharmaceutical companies, research institutions, and investors. This creates a supportive ecosystem that sustains momentum across short‐, medium‐, and long‐term goals, ultimately advancing toward the vision of curing rare diseases.

***Exploration**:* Translational medicine is a highly popular concept today, but in practice, how can funders and researchers build mutual trust and achieve shared success? From laboratory to CRO to pharmaceutical companies, at what point in the research process do you believe transferring laboratory results can most effectively accelerate translation into medicine? Are there proven business models to draw from? What is your team's strategy?

**Mr. Cai Lei and Ice‐Breaking Team**:

Translational medicine, as the bridge between basic science and clinical application, has received increasing attention in recent years. Yet the success or failure of this process often depends on whether funders and researchers can establish trust and work toward shared goals. Bridging the gap between academic inquiry and industrial application is not only a scientific issue but also a real‐world challenge that directly impacts patients' well‐being.

Building trust begins with transparency and communication. Funders, whether government, foundations, or industry, require clear expectations of return, while researchers must respond with rigorous data and openness. Regular progress sharing and clearly defined agreements on intellectual property and publication rights form the foundation for bridging differences. The SPARK program at Stanford University exemplifies this: by providing a transparent collaboration framework, it has successfully accelerated laboratory discoveries into the clinic. In reality, conflicts of interest and resource asymmetries often cloud cooperation. Long‐When Rare Is Not Small: Amyotrophic Lateral Sclerosis initiatives and therapy? term partnerships provide an antidote; Pfizer's Centers for Therapeutic Innovation, for example, operate by sharing risks and resources, speeding up development while preserving academic independence.

Shared success requires both sides to move beyond their comfort zones. Funders should provide not only financial backing but also technical support, market insights, and policy leverage, for instance, tax incentives for rare disease research. Researchers, for their part, must actively align with clinical needs, such as identifying measurable biomarkers early in the process. Most importantly, patients must be placed at the heart of collaboration. Through co‐design models, patient needs can guide research direction and offer both funders and researchers a unifying mission, improving quality of life, rather than chasing profit or publication alone.

From the perspective of patient organizations like ours, saving lives is the ultimate goal, not simply commercial returns or academic recognition. Based on this principle, funders and researchers must set up regular and ad hoc communication mechanisms to ensure timely and accurate information flow. Funders gain clear visibility into project progress, challenges, and resource requirements, while researchers gain a better understanding of funders' expectations, risk tolerance, and investment plans. Detailed contracts are also essential, covering the use of funds, ownership of outcomes, benefit‐sharing, and liability in case of breach.

In the ALS field, our team plays a dual role, as both funder and researcher. We actively collaborate with diverse institutions and scientists to expand the research network and foster cross‐disciplinary integration across proteomics, metabolomics, immunomics, genomics, and spatial‐temporal omics. This accelerates the transition from basic ALS research to clinical application.

Our strategic model for translational projects follows four steps:

1. Discovery of therapeutic leads: Our team identifies candidate approaches by systematically screening global scientific literature and monitoring emerging developments.
2. Partnering and negotiation: We directly lead discussions with collaborators, organizing frequent meetings, both virtual and in‐person, and handling contract drafting, negotiations, and revisions.
3. Preclinical validation: Third‐party laboratories perform in vitro and in vivo studies to assess safety and efficacy, with our team sending staff to be embedded on‐site.
4. Clinical translation: Once conditions are met, we advance to toxicology, pharmaceutical formulation, and delivery optimization, and then use rapid recruitment platforms to launch clinical trials in partnership with external CROs.

In summary, the future of translational medicine depends on establishing a culture of trust and mutual achievement between funders and researchers. When collaboration shifts from negotiation to co‐creation, laboratory discoveries can truly be translated into clinical hope. Looking forward, policy incentives and technological advances, such as AI‐driven drug screening, will further accelerate progress. Our vision is to help build a transparent, patient‐centered translational ecosystem where trust, innovation, and collaboration converge to bring real benefits to patients.

## Section 3: Research Ecosystem and Multi‐Stakeholder Collaboration

***Exploration**:* Our peers deeply respect these efforts and believe that overcoming ALS requires more leaders in the spirit of “Cai Lei”. From the perspective of employment and career development, how can we encourage young scholars to dedicate themselves to rare disease research? Could joint special funds or industry–academia–research training programs be established in collaboration with government bodies that manage policy and funding specifically for science and technology, or education authorities?

**Mr. Cai Lei and Ice‐Breaking Team**:

We believe that motivating young scholars to enter the rare disease field requires a combination of stable funding, collaborative training, long‐term career pathways, and enhanced social recognition.

1. Establish dedicated funding mechanisms:

Joint Special Funds for Rare Disease Research: In collaboration with the National Natural Science Foundation of China, the Ministry of Science and Technology, and provincial science and education departments, special funds should be set up. National‐level agencies could provide overarching planning and prioritize frontier research, while local authorities could supply targeted resources aligned with local scientific strengths and specific rare disease needs. This ensures young researchers have a stable source of funding.

Expansion of the “Ice‐Breaking Scholarship:” The Ice‐Breaking Scholarship could be formalized with rigorous evaluation criteria to recognize outstanding young scholars who achieve breakthroughs in ALS and other rare diseases. Wider promotion of such awards would not only highlight role models but also attract more talent into the field.

2. Promote industry‐academia‐research training models:

Integrated training programs: Build cooperative training frameworks linking universities, research institutes, and pharmaceutical companies. Universities can provide theoretical education, research institutes can offer experimental platforms, and industry can provide practical experience. This allows young scholars to gain full‐process exposure from early‐stage R&D to clinical translation.

Strengthening international exchange: Encourage participation in global conferences and joint projects on rare diseases, enabling young researchers to access frontier technologies, broaden perspectives, and improve competitiveness.

3. Optimize career development pathways:

Long‐term positions: Establish permanent faculty positions dedicated to rare disease research within universities and institutes, with clear evaluation metrics, providing young scholars with security and motivation for long‐term commitment.

Talent pool and career guidance: Build a rare disease research talent database, monitor career development, provide professional mentorship, and create bridges to potential employers, facilitating effective career progression.

4. Enhance social awareness and recognition:

Public engagement and science communication: Use media and science outreach to highlight advances in rare disease research and showcase the contributions of young scholars. This raises public awareness and enhances researchers’ sense of professional honor.

Involvement in public welfare: Organize opportunities for young scholars to participate in patient‐centered activities. Direct engagement with patients fosters a deeper sense of mission while also demonstrating the value of rare disease research to society, helping to inspire more talent to join the field.

In summary, a supportive ecosystem combining financial security, cross‐sector training, long‐term career incentives, and societal recognition is key to inspiring the next generation of researchers to dedicate themselves to rare diseases such as ALS.

***Exploration**:* The priorities of philanthropic organizations, patient families, and pharmaceutical companies are not always aligned. You have experimented with livestream shopping and joint laboratory models to reconcile these differences. Do you think these experiences could be applied more broadly across the rare disease field? And how might other rare disease communities foster their own sources of hope, comparable to the role you have played in ALS?

**Mr. Cai Lei and Ice‐Breaking Team**:

Conflicting interests are a constant reality in the rare disease space. Patients and families place survival and access to treatment above all else, while companies must consider business sustainability and long‐term growth. This contradiction is particularly acute in rare diseases. Many of these conditions, especially those caused by single‐gene mutations or missing proteins, could in principle be treated with technologies such as gene therapy. In practice, patients are left with few effective options, and companies often lack the funds to advance promising ideas. It is worth noting that responsibility for early‐stage development, from scientific concept through initial clinical trials, usually rests with smaller biotech ventures rather than global pharmaceutical firms like Pfizer or Merck. These smaller companies are typically led by capable scientists, but without revenue‐generating products, they depend heavily on outside investment.

To break through this dilemma, we explored new approaches. Livestream shopping has raised public awareness of rare diseases while directly supporting preclinical studies, moving potential treatments forward more quickly. In parallel, we established a nationwide joint laboratory network with leading scientists, research institutes, and innovative biotech companies. This collaboration brings together expertise in target discovery, drug development, multi‐omics, stem cell‐based disease modeling and screening, traditional medicine, and clinical translation. The network consolidates resources, accelerates innovation, and helps research move toward the clinic. One example is our ALS joint laboratory with Ractigen Therapeutics. Beyond research, we have also focused on public engagement. We produced documentaries on projects such as RAG‐17, shared progress widely through media platforms, and invited figures such as Dr. Longcheng Li, CEO of Ractigen Therapeutics, to take part in our livestream selling sessions. These initiatives have given patients, particularly those with *SOD1* and *FUS* mutations, greater knowledge and confidence that their disease can be tackled. They have also drawn wider social attention to the scientists and companies involved, creating a more supportive environment for future collaboration and investment.

Our long‐term goal is to turn the ALS experience into a transferable model for other rare diseases. The challenges, however, go beyond limited visibility. They also include weak patient organization and chronic underfunding. What is needed is not one figure like “Cai Lei,” but many. Given the small size of each rare disease community, no individual can carry the burden alone. Only when awareness is widely shared, and patients, scientists, and partners work together, will more sources of hope continue to emerge.

***Exploration**:* The “Life Sciences Ice‐Breaking Award” you initiated has received wide attention. Could it evolve into a global collaborative network for research? And might it also begin to encourage participation from undergraduate students, thereby fostering early interest in rare disease research?

**Mr. Cai Lei and Ice‐Breaking Team**:

To inspire scientific talent, in 2024, we launched two initiatives: the “Life Sciences Ice‐Breaking Award” and the “Ice‐Breaking Scholarship.” The Life Sciences Ice‐Breaking Award recognizes scientists, clinicians, biopharmaceutical companies, and technology enterprises that have made outstanding contributions to ALS research and, most importantly, to saving lives. As the first life sciences prize established and conferred by a patient‐led organization, its core criterion is whether research outcomes translate into tangible benefit for patients, rather than academic prestige alone.

In parallel, the Ice‐Breaking Scholarship was created to encourage a new generation of researchers to engage in ALS and other neurodegenerative diseases such as Alzheimer's disease and Parkinson's disease. The program supports promising individuals at all stages—from undergraduates to postdoctoral fellows—providing recognition and resources that can spark sustained interest in rare disease research.

Looking ahead, our aspiration is to expand the Life Sciences Ice‐Breaking Award into a global platform for scientific collaboration. We envision a network where recognition is not limited by geography, but instead guided by a single principle: genuine contributions to saving lives. By opening the award to achievements worldwide, we hope to bring together the best ideas and capabilities across borders, mobilize collective intelligence, and accelerate progress in rare disease research. In this way, the award is not merely a symbol of honor but also a catalyst for building an international community dedicated to delivering hope for patients.

***Exploration**:* Rare diseases are frequently perceived as commercially unattractive. How can multinational pharmaceutical companies be persuaded to enter this sector?

**Mr. Cai Lei and Ice‐Breaking Team**:

In Believe (Mr. Cai's biography), I wrote: “When you seek to mobilize others, it is not enough to state your own needs. You must also demonstrate what you can offer them.” This principle applies directly to rare disease drug development. Appeals to compassion alone cannot sustain pharmaceutical investment. What drives companies is the prospect of viable returns. If orphan drug prices are forced too low, it may seem to expand patient access in the short term, but in reality, it removes the incentive for companies to develop such therapies. The result is that patients may end up with no medicines at all. This is why orphan drug pricing protections are critical: they ensure that innovation is rewarded, which in turn makes new therapies possible. Once drugs are available, affordability should be addressed through mechanisms such as public health insurance, commercial insurance, or subsidies. The case of spinal muscular atrophy treatments illustrates this balance, where appropriate pricing, combined with reimbursement systems, enabled both innovation and patient access.

The deeper challenge lies in the structure of rare disease markets. Patient populations are small, and most families face significant financial constraints. As a result, the risks and costs of R&D are disproportionate to the potential return. Drug development is inherently a business activity, and without mechanisms to share those costs, companies and investors are unlikely to commit resources. At present, much of the engagement of multinational pharmaceutical companies in rare diseases remains within the realm of corporate responsibility rather than commercially sustainable strategy.

To change this dynamic, rare disease research must be positioned not as an isolated burden but as a shared responsibility. This requires frameworks in which patients, governments, insurers, and industry collaborate to distribute risks and create room for sustainable innovation. In China, especially, it is vital to articulate both the needs and the capacities of rare disease communities, while exploring innovative models of reimbursement and partnership. Only then can global pharmaceutical companies see rare disease drug development not as “unprofitable,” but as an achievable and meaningful endeavor—one where scientific innovation and patient benefit are aligned.

## Section 4: Patient Cases and Ethical Dilemmas

***Exploration**:* Social support and ethical challenges are critical dimensions of rare disease research. Issues such as the ethical boundaries of gene editing, access to imported medicines, and models of collaboration are frequently debated. How can these tensions be addressed, and what broader reflections do you have for academic and public audiences worldwide?

**Mr. Cai Lei and Ice‐Breaking Team**:

ALS is a profoundly heterogeneous disease; no two patients progress in the same way. On February 27, 2025, the death of 26‐year‐old patient Jingwen Chen, widely reported online, drew renewed attention to the rare disease community. Another patient with the same *SOD1* mutation was fortunate to enroll in an investigator‐initiated trial (IIT), which slowed his disease. Jingwen, however, deteriorated so rapidly that by the time a relevant trial opened, she no longer met the entry criteria. Despite accelerated efforts by hospitals, companies, and advocates, the treatment arrived too late for her. Such tragedies, echoed in earlier cases like that of Tao Lou, an extraordinary graduate from Peking University, highlight the ethical urgency of narrowing the gap between scientific progress and patient access.

### Drug Development and Economic Burden

Important advances have been made. Tofersen, the first therapy to target an ALS‐causing gene, marked a milestone, yet its annual cost of roughly 1.4 million RMB places it beyond reach for most families. RAG‐17, a small interfering RNA (siRNA) therapy co‐developed with Ractigen Therapeutics, is showing promising early signals in phase I trials. Still, with average survival measured in only three to five years, patients cannot afford to wait for lengthy approval timelines.

### Law Versus Patient Rights

Patients often voice a simple but profound appeal: “Mr. Cai, is there any way I could try the drug? Even if it has not yet entered clinical trials, I am not afraid of dying, I only fear waiting to die.” In the absence of approved treatments, many patients are seeking options abroad or turn to experimental interventions through clinical trials. For many, this becomes an act of self‐preservation, yet the journey is often arduous and the timelines painfully uncertain. Yet the law also has a duty to safeguard public health. The central question is how to uphold regulatory authority while also responding to the extraordinary challenges faced by rare disease patients. Lawful but compassionate “expanded access pathways” are needed to balance these priorities.

### National Policy Support

In fact, China has already moved in this direction. Since 2014, legal interpretations have exempted non‐profit, patient‐initiated access to medicines from criminal liability. Regulatory reforms have followed:

- From 2016 onward, rare disease drugs have been eligible for priority review, and since 2018, more than 50 urgently needed overseas therapies have been placed on fast‐track lists, with 26 already approved domestically.
- In 2020, revised regulations formalized accelerated timelines, while new State Council guidelines in 2024 shortened review periods for innovative drugs to as little as 30 working days, the fastest in any category.
- Requirements for registration testing and clinical trials have been eased for qualifying rare disease products, reducing both time and cost.
- Tax incentives and tariff exemptions since 2019 have lowered the burden of importing or developing orphan drugs, and draft regulations now propose up to seven years of market exclusivity.
- In 2024, the “Care Program (关爱计划)” was launched to promote patient‐centered trial design for rare diseases.

Despite these reforms, the demand for life‐saving treatments remains pressing, particularly for severe and intractable diseases where conventional therapies offer little benefit. Cell and gene therapies, which have become a global priority in biomedicine, show extraordinary promise in such areas. In China, however, their development and translation still face critical hurdles. A central barrier is that data generated from IITs cannot be applied directly in clinical practice. Instead, they must be converted into industry‐sponsored trials (ISTs) and then undergo the full registration process before patients can receive treatment. While this pathway safeguards rigor, it also creates delays that, for patients with rapidly progressing diseases such as ALS can mean the difference between access and loss of opportunity.

### International Experience

Other jurisdictions offer valuable lessons for balancing safety with timely access. In the United States, IITs are supported by a wide range of sponsors, academic centers, CROs, and patient organizations, and benefit from flexible ethics review and risk‐based classification. This diversity broadens innovation and enables earlier insights that can feed into formal development. Europe has adopted a “hospital exemption” framework, allowing advanced therapies to be used in urgent cases outside full market authorization, while sustaining a strong tradition of non‐commercial clinical research. Japan has pioneered conditional approvals for regenerative medicine products, permitting their use based on early data from IITs while requiring rigorous post‐marketing evidence. These mechanisms do not bypass safety, but they do acknowledge that patients with life‐threatening diseases cannot wait for the traditional pace of drug development.

### Outlook

Our vision is of a rare disease research community functioning as an “infinite arsenal,” a shared reservoir of knowledge, resources, and commitment. Realizing this requires not only science but also legal flexibility, ethical imagination, social solidarity, and international collaboration. To both academic readers and the wider public, our message is clear: progress against rare diseases cannot rest on a few shoulders. Only through collective effort can we transform despair into hope.

## Editorial Note

### A Call to Action: Reframing Rare Disease Innovation as Shared Responsibility


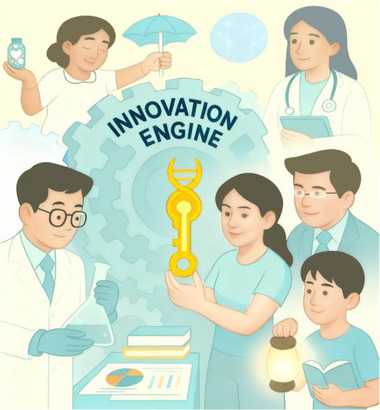


The future of rare disease treatment will not be unlocked by science alone; it requires alignment across a fragmented ecosystem.

For scientists: Rare diseases are not peripheral curiosities, but central to advancing personalized medicine. They offer tractable genetic models, rapid translational potential, and opportunities to pioneer platform technologies. What is lacking is not complexity, but sustained commitment.

For industry: The retreat from AAV reflects real risks, but also a missed opportunity. Rare disease pipelines demand new models, modular platforms, adaptive trials, and cost‐sharing consortia, not traditional blockbuster logic. Strategic patience and precision will define long‐term leadership.

For funders: High‐impact innovation often starts where market logic fails. Patient‐led trials, translational registries, and early‐stage proof‐of‐concept studies are under‐resourced but critical. Risk‐sharing mechanisms and flexible grants must be expanded to close this gap.

For clinical investigators: Rare disease trials require agility, not volume. Adaptive design, biomarker‐based stratification, and decentralized recruitment through patient networks like AskHelpU are no longer optional, they are the path forward.

For international collaborators: Regulatory fragmentation, data silos, and slow ethics approvals are solvable problems. Shared protocols, global biobanks, and patient registries are essential infrastructure for current translational medicine.

For patients: Your voices are no longer passive. As partners, funders, and even trial initiators, patient communities are reshaping biomedical innovation—from driving research to advocating for a whole life‐cycle care system that integrates diagnosis, treatment, rehabilitation, and social support. The model built by Mr. Cai and his team shows that urgency, agency, and collaboration can accelerate progress where conventional systems stall.

For the public: Rare does not mean irrelevant. Rare diseases illuminate how we value life, equity, and scientific investment. Supporting rare disease innovation is not charity, it is a litmus test for how future‐ready our healthcare systems truly are.

## Author Contributions

Y.L., X.X., Y.C., H.Y., and X.C. contributed equally to this manuscript. Y.L. drafted the manuscript and initiated the interview. Y.L., X.X., X.C., and H.Y. participated in data compilation, figure design, literature analysis, and manuscript revising and editing. Y.C. composed the wording of the interview on behalf of L.C. L.C. provided critical insight, patient engagement perspectives, and contributed to the interview content. B.S. made inputs on structure, scientific opinion, and revision. All authors reviewed and approved the final manuscript.

## Funding

This study was supported by the China National Postdoctoral Program for Innovative Talent, China Postdoctoral Science Foundation (BX20240103, 2025M780051), the Henan Provincial Medical Science and Technology Research Program (Joint Provincial‐Ministerial Project SBGJ202503048), and the Henan Provincial Health Commission.

## Conflicts of Interest

The authors declare no conflicts of interest. Author information is fully disclosed. Mr. Cai Lei is the founder of AskHelpU, a patient advocacy and research platform mentioned in this article. His contributions in this manuscript were sharing perspectives and factual information relevant to patient‐led innovation. No commercial, financial, or proprietary relationships exist that could be construed as a potential conflict of interest. Y.L., X.X., and B.S. are the editors at *Exploration*.
